# Loss and recovery of myocardial mitochondria in mice under different tail suspension time: Apoptosis and mitochondrial fission, fusion and autophagy

**DOI:** 10.1113/EP090518

**Published:** 2023-08-11

**Authors:** Zhe Wang, Xing‐Chen Wang, Ya‐Fei Chen, Chuan‐Li Wang, Le Chen, Ming‐Yue Jiang, Xi‐Wei Liu, Xiao‐Xuan Zhang, Yong‐Zhen Feng, Jin‐Hui Xu

**Affiliations:** ^1^ College of Life Sciences Qufu Normal University Qufu Shandong China

**Keywords:** apoptosis, heart, microgravity, mitochondria

## Abstract

Long‐term weightlessness in animals can cause changes in myocardial structure and function, in which mitochondria play an important role. Here, a tail suspension (TS) Kunming mouse (*Mus musculus*) model was used to simulate the effects of weightlessness on the heart. We investigated the effects of 2 and 4 weeks of TS (TS2 and TS4) on myocardial mitochondrial ultrastructure and oxidative respiratory function and on the molecular mechanisms of apoptosis and mitochondrial fission, autophagy and fusion‐related signalling. Our study revealed significant changes in the ultrastructural features of cardiomyocytes in response to TS. The results showed: (1) mitochondrial swelling and disruption of cristae in TS2, but mitochondrial recovery and denser cristae in TS4; (2) an increase in the total number of mitochondria and number of sub‐mitochondria in TS4; (3) no significant changes in the nuclear ultrastructure or DNA fragmentation among the two TS groups and the control group; (4) an increase in the bax/bcl‐2 protein levels in the two TS groups, indicating increased activation of the bax‐mediated apoptosis pathway; (5) no change in the phosphorylation ratio of dynamin‐related protein 1 in the two TS groups; (6) an increase in the protein levels of optic atrophy 1 and mitofusin 2 in the two TS groups; and (7) in comparison to the TS2 group, an increase in the phosphorylation ratio of parkin and the ratio of LC3II to LC3I in TS4, suggesting an increase in autophagy. Taken together, these findings suggest that mitochondrial autophagy and fusion levels increased after 4 weeks of TS, leading to a restoration of the bax‐mediated myocardial apoptosis pathway observed after 2 weeks of TS.

## Abstract (highlights)

- **What is the central question of this study?**
  What are the effects of 2 and 4 weeks of tail suspension on myocardial mitochondrial ultrastructure and oxidative respiratory function and on the molecular mechanisms of apoptosis and mitochondrial fission, autophagy and fusion‐related signalling?
- **What is the main finding and its importance?**
  Increased mitochondrial autophagy and fusion levels after 4 weeks of tail suspension help to reshape the morphology and increase the number of myocardial mitochondria.

## 1 INTRODUCTION

Earth‐based simulation of the physiological effects induced by weightless spaceflight is crucial in space biology and medicine (Perhonen et al., 2001). Animal tail suspension (TS) models are widely used to simulate the effects of weightlessness, inducing hindlimb musculature atrophy, a chronic increase cardiac in volume load and a reduction in plasma volume after only 7 days (Brizzee & Walker, 1990). The volume of myocardial cells decreases, and local muscle fibre disorders and nuclei disappear after only 2 weeks of TS (Kim, 2012). Furthermore, cytochrome *c* levels in the protein membrane mitochondrial fraction increase (by 34.6%) in the myocardium after only 18 h of TS (Ogneva et al., 2014). Therefore, investigating mitochondria in the ventricular cardiomyocytes of animals under TS is important because the changes in mitochondrial homeostasis are non‐specific adaptive responses to changes in myocardial functioning conditions (Lipina et al., 2003).

Mitochondria are the major sites of aerobic oxidation and ATP energy supply in cells (Dugbartey et al., 2017; Kramarova et al., 2008). Although the impact of long‐term TS on oxidative respiration‐related factors in myocardial mitochondria remains unclear, studies have reported a significant reduction in mitochondrial oxidative respiratory function in rat skeletal muscles and cerebral arteries after 4 weeks of TS (Yajid et al., 1998; Zhang et al., 2014). Compared with skeletal muscle, mitochondria‐related genes in the rat myocardium show significant transcriptional changes in microgravity conditions (Connor & Hood, 1998). Proteomic analysis has also shown that exposure to microgravity impacts myocardial mitochondria‐related protein levels (Feger et al., 2016). However, whether the loss of myocardial mitochondria in TS mice affects mitochondrial oxidative respiratory function remains unclear.

Changes in the morphology and number of mitochondria are closely related to the dynamic balance among apoptosis, mitochondrial fission, fusion and autophagy (Tilokani et al., 2018; Yamano et al., 2016). Studies have shown that levels of apoptosis in myocardial fibres are increased after 8 weeks of TS (Sun et al., 2019), which is related to an increase in the ratio of bcl‐2‐associated X protein (bax) to B‐cell lymphoma/leukemia‐2 (bcl‐2) (Chang et al., 2015).

Cell mitochondria are in a state of dynamic balance, with continuous maintenance through mitochondrial fission, fusion and autophagy (Kane et al., 2014; Kraus & Ryan, 2017). Mitochondrial fission and fusion are opposing processes that maintain the mitochondrial network in living cells and ensure normal function, in conjunction with autophagy (Gilkerson et al., 2021; Griparic et al., 2007; Michalska et al., 2016; Rujiviphat et al., 2009). Although research on the myocardium in weightless conditions is limited, existing studies have shown that the protein levels of mitofusin 1 and 2 (MFN1 and MFN2) and dynamin‐related protein 1 (DRP1) are significantly reduced in the tibialis anterior and gastrocnemius muscles of mice after 1 and 4 weeks of TS, suggesting weakened mitochondrial fission and fusion (Cannavino et al., 2015; Liu et al., 2012). Therefore, further investigations of mitochondrial fission, fusion and autophagy‐related signalling should help to elucidate the mechanisms underlying changes in mitochondria during simulated weightlessness.

Based on the above, we hypothesized that the morphology, quantity and oxidative respiratory function of myocardial mitochondria would change in mice subjected to TS for different periods. We also hypothesized that these changes would be related to changes in apoptosis and mitochondrial fission, fusion and autophagy. To test these hypotheses, we examined the ultrastructure of the myocardium in mice after different periods of TS [control (CON), 2 weeks (TS2) and 4 weeks (TS4)] and analysed protein levels and the apoptosis and mitochondrial function, fission, fusion and autophagy‐related signalling activities. Moreover, we explored the underlying molecular mechanisms associated with the effects of TS on myocardial mitochondrial structure and function. Our findings might provide insights into the adaptive changes that occur in the human heart during simulated microgravity.

## 2 MATERIALS AND METHODS

### 2.1 Ethical approval

All experiments conducted conformed to the principles set out by Grundy (2015) and the ARRIVE guidelines and were approved by the Biomedical Ethics Committee of Qufu Normal University (permit number: dwsc 2021052).

### 2.2 Animals and groups

Kunming mice (*Mus musculus*) were purchased from Pengyue Experimental Animal Breeding (Jinan, China). The mice were housed, two per cage (28 cm × 18 cm × 12 cm), at an ambient temperature of 22 ± 2°C, relative humidity of 55 ± 5% and light regime of 12 h–12 h light–dark (light on from 06.00 to 18.00 h). Standard mouse chow (Pengyue Experimental Animal Breeding, China) and water were provided ad libitum; sterilized poplar wood shavings (Pengyue Experimental Animal Breeding) were used as bedding and cardboard tubes as enrichment. Poplar wood shavings were replaced every 3 days to ensure that the animals were in a comfortable living environment. During a 2 week acclimation period, the mice were allowed to move freely and become accustomed to the laboratory conditions. To ensure adequate nutrient intake (3–9 g), 20 g of food was provided daily, and daily food consumption was recorded. The volume of the provided water bottle was 500 mL, and the purified water was refreshed daily to ensure that animals received sufficient drinking water. Resource inspections were conducted once per day.

Upon completion of the adaptation period, all mice were numbered, weighed and randomized into three groups (*n* = 16), one per cage. The mice were 7 weeks old at the beginning of the formal experiments and were housed individually in suspended tail experimental boxes. Daily food and water intake were recorded for each mouse. The suspended tail procedure was performed as described previously (Hu et al., 2017), using the Morey‐Holton method (Morey‐Holton & Globus, 2002). Briefly, the tail was cleaned, sterilized and covered with orthopaedic tape‐adhesive plaster (colophony), wrapped with pervious gauze and half‐tied with tape. The tail was subsequently connected to a swivel joint at the top of the cage using tape, enabling 360° rotation to ensure unrestricted movement for the animal. The suspension cages measured 30 cm × 35 cm × 45 cm, and the procedure was performed without restricting the movement of the mice or damaging the tail tissue. The three groups were as follows: control (CON) group, in which mice were free to move for 4 weeks; 2 week TS group (TS2), in which mice were suspended by their tails at a 30° angle (between the body and the horizontal plane) for 2 weeks after 2 weeks of free movement; and 4 week TS group (TS4), in which mice were suspended by their tails for 4 weeks. All groups were maintained in the same environmental conditions, including light, temperature and humidity, and all mice were 11 weeks old at the end of the treatment period.

### 2.3 Sample preparation

At the end of the experimental period, all mice were killed by CO_2_ asphyxiation at 08.00 h. The mice, together with their respective cages, were placed in a sealed container and exposed to 99% CO_2_ at a flow rate of 50% volume/min for 5 min to ensure humane killing. The body weight (BW), body length (BL), carcass weight (CW; body mass after removal of the viscera), tibia length (TL) and myocardial mass (MM) of the mice were recorded. The myocardium of eight mice from each group was frozen with liquid nitrogen and stored at −80°C for western blot and protein activity analysis. The myocardium of the other mice was used for transmission electron microscopy and frozen section experiments. All procedures were carried out in accordance with approved guidelines.

### 2.4 Transmission electron microscopy

Cardiac muscle was examined via transmission electron microscopy (Hitachi, HT7800, Japan), as described previously (Biazik et al., 2015; Wang et al., 2019). The ultrastructure of the nucleus was observed at ×5000 magnification. Mitochondrial density within a defined region (10 μm × 10 μm area) was determined at ×3000 magnification, and mitochondria were distinguished at ×7000 magnification. Sub‐mitochondria were counted in a randomly selected area (10 μm × 10 μm) at the edge of the myocardial fibres at ×3000 magnification. Mitochondrial edges were drawn using NIH Image‐Pro Plus v.6.0 at ×3000 magnification. Mitochondrial area and total muscle fibre area were determined in a randomly selected region (10 μm × 10 μm) to obtain the ratio of mitochondria to total area.

### 2.5 Terminal deoxynucleotidyl transferase biotin‐dUTP nick end‐labelling staining

DNA fragmentation induced by apoptosis was determined by double‐labelled fluorometric terminal deoxynucleotidyl transferase biotin‐dUTP nick end‐labelling (TUNEL), as described previously (Kong et al., 2020). In short, frozen tissue was cut into 10‐μm‐thick sections and stained using a TUNEL Staining Kit (#MK1023; Boster, Wuhan, China). 4′6′‐Diamidino‐2‐phenylindole (DAPI; 1:100, catalogue no. D1306; Sigma‐Aldrich, Saint Quentin Fallavier, France) staining was used to count the number of nuclei. Positive and negative controls were included in each experiment. Sections were treated with DNase I (Tiangen, Beijing, China) in DNase buffer for 10 min at room temperature before incubation with the TUNEL reaction mix (positive control) or without the TdT enzyme (negative control) for 30 min at 37°C. Images were visualized using a confocal laser scanning microscope (Olympus, Osaka, Japan) at an objective magnification of ×40.

### 2.6 ATP synthase, citrate synthase and caspase 3 activity

Samples stored at −80°C were used to detect ATP synthase, citrate synthase (CS), and caspase 3 activity.

ATP synthase activity was determined by measuring the free phosphate group at 450 nm using an ATP Synthase Activity Assay Kit (BC0065; Solarbio, Beijing, China) according to the manufacturer's instructions (Chen et al., 2019). Standard phosphorus stock solution (10 μmol/mL) was used. ATP synthase activity was determined by comparing the absorbance of the tested sample with that of the standard at 450 nm.

The CS activity was determined by measuring coenzyme A (CoA) formation at 450 nm with a Citrate Synthase Activity Assay Kit (BC1060, Solarbio) according to the manufacturer's instructions (Zhang et al., 2020). Citrate synthase catalyses acetyl‐CoA and acetoacetic acid to generate citryl‐CoA, which is hydrolysed further to generate citric acid. This reaction promotes the transformation of colourless 5,5′‐dithiobis‐(2‐nitrobenzoic acid) (DTNB) and 2‐nitro‐5‐thiobenzoic acid (TNB), which shows absorbance at 412 nm. One unit of enzymatic activity is defined as the amount of enzyme that catalyses the production of 1 nmol of TNB per minute per milligram of tissue protein at 37 or 25°C.

Here, caspase 3 activity in the cell lysates was determined using a Caspase 3 Activity Kit (BC3830, Solarbio, Beijing, China) following the manufacturer's protocols (Wang et al., 2020). This colorimetric assay is based on the hydrolysis of the peptide substrate Asp‐Glu‐Val‐Asp‐*p*‐nitroaniline (DEVD‐pNA) by caspase 3, which results in the release of the pNA moiety characterized by high absorbance at 405 nm. Thus, caspase 3 activity can be calculated by detecting pNA and comparing it with the 5 mM pNA standard.

### 2.7 Western blotting

Frozen tissue samples (0.1 g) were homogenized with RIPA lysis buffer. The soluble protein concentration was determined using a Pierce BCA Protein Quantification Kit (#53225; Thermo Fisher Scientific, USA). The samples were then adjusted to a final protein concentration of 3 μg/μL with 1× SDS loading buffer (#1112; Boster, China) and homogenizing buffer. The final protein samples were stored at −80°C until further use.

Equal amounts of protein from each sample (20 μL) were loaded onto 10% polyacrylamide gels containing 0.5% trichloroethanol, with electrophoresis run at 120 V for 60–100 min (Ladner et al., 2004). Fluorescence images of gels were captured using an electrophoresis gel imaging analysis system (Bio‐Rad, CA, USA) and total protein levels were analysed. The proteins were then transferred electrically to polyvinylidene fluoride membranes (0.45 μm pore size) using a Bio‐Rad wet transfer apparatus (Kurien & Scofield, 2015). The blotted membranes were blocked with 5% skimmed milk powder in Tris‐buffered saline (TBS; 150 mM NaCl, 50 mM Tris‐HCl, pH 7.5) and incubated with rabbit‐derived primary antibody (Table 1) in TBS containing 0.1% bovine serum albumin at 4°C overnight. The membranes were then incubated with IRDye 800 CW goat‐anti rabbit secondary antibodies (1:5000 dilution; #31460; Thermo Fisher Scientific, USA) for 90 min at room temperature and visualized with an Odyssey scanner (Bio‐Rad).

**TABLE 1:** Primary antibodies used in western blot analysis.

Protein name or symbol	Details of antibodies	References
ATP synthase	1:1000, #14676, Proteintech, Wuhan, China	Schäfer et al. (2022); Ugbode et al. (2020)
CS	1:1000, #16131, Proteintech	Babetto et al. (2020); Fong et al. (2015)
Bax	1:1000, #50599, Proteintech	Dai et al. (2018); Zierhut et al. (2019)
Bcl‐2	1:1000, #3498, Cell Signaling Technology CST, Danvers, MA, USA	de Melo Gomes et al. (2023)
DRP1	1:1000, #12957, Proteintech	Guo et al. (2020); Meng et al. (2022)
Phospho‐DRP1	1:1000, #AF8470, Affinity Biosciences, OH, USA	Zeng et al. (2019)
MFF	1:1000, #17090, Proteintech	Franco et al. (2016); Lee et al. (2016)
Parkin	1:1000, #14060, Proteintech	Aras et al. (2020); Davis et al. (2021)
Phospho‐parkin	1:1000, AF3500, Affinity Biosciences	Abudureyimu et al. (2020)
PINK1	1:1000, #23274, Proteintech	Elswood et al. (2021); Xu et al. (2022)
OPA1L	1:500, #19645R, Bioss, Beijing, China	–
MFN1	1:1000, #13798, Proteintech	Brahmachari et al. (2019); Sharoar et al. (2016)
MFN2	1:1000, #12186, Proteintech	Jiao et al. (2021); Sharoar et al. (2016)
Caspase 3	1:1000, #19677, Proteintech	Shen et al. (2022)
LC3	1:2000, #14600, Proteintech	Ho et al. (2019); Sen et al. (2022)
P62	1:5000, #18420, Proteintech	Inoue et al. (2023); Wan et al. (2018)

The fold‐change of each target protein relative to the CON levels was calculated. The immunoblot band density in each lane was standardized against the summed intensity of total protein. Enhanced chemiluminescence (ECL) band intensities were analysed using Image‐Pro Plus v.6.0 software (Media Cybernetics, Rockville, MD, USA) as in previous studies (Gilda & Gomes, 2013; Gürtler et al., 2013; Ladner et al., 2004; Vigelsø et al., 2015). A sample from each gel was selected as the standard (e.g., the first sample of each gel, i.e., samples 1, 3, 5 and 7 of the control group) for ECL and gel comparison analyses to control against protein loading irregularities. The use of gels containing protein loading controls was preferred over a single housekeeping gene for total protein normalization, because it has been shown to be more effective in natural model systems (Wijenayake et al., 2017; Xu et al., 2021).

### 2.8 Statistical analyses

All data were analysed using SPSS v.22.0 and presented as the mean ± SD. Overall and group differences were determined using one‐way ANOVA and Fisher's least significant difference post hoc test, respectively. In circumstances where no homogeneity was detected, ANOVA–Dunnett's T2 method was applied. Results were considered significant at *P* < 0.05.

## 3 RESULTS

### 3.1 Mouse body size and proportions

No significant differences in BW were observed among the three groups before the experiment. After 4 weeks of TS treatment, the BW, BL and CW values were higher in the CON group than in the TS2 and TS4 groups, suggesting that TS treatment significantly inhibited body growth (*P* < 0.001). However, the MM/TL, MM/BW and MM/CW ratios were highest in the TS4 group and lowest in the TS2 group (*P* < 0.001; Table 2).

**TABLE 2:** Effects of tail suspension on mouse body size and proportions.

Parameter	CON	TS2	TS4
BW before treatment (g)	31.22 ± 1.61	31.02 ± 1.59	31.32 ± 1.63
BW after treatment (g)	43.54 ± 3.89^a^	38.56 ± 2.87^b^	36.88 ± 3.39^b^
BL after treatment (g)	11.23 ± 0.52^a^	10.16 ± 0.44^b^	10.11 ± 0.51^b^
CW after treatment (g)	28.86 ± 2.86^a^	24.39 ± 2.01^b^	24.99 ± 1.90^b^
MM (g)	0.257 ± 0.053^a^	0.200 ± 0.028^b^	0.236 ± 0.048^a^
TL (cm)	2.02 ± 0.10^b^	2.08 ± 0.13^a^	2.12 ± 0.08^a^
MM/TL (g/cm)	0.128 ± 0.028^a^	0.096 ± 0.014^c^	0.110 ± 0.023^b^
MM/BW (mg/g)	5.90 ± 0.92^a^	5.20 ± 0.52^b^	6.37 ± 1.02^a^
MM/CW (mg/g)	8.93 ± 0.93^ab^	8.25 ± 1.10^b^	9.49 ± 0.98^a^

*Note*: Values are means ± SD (*n* = 16). Different superscript letters indicate significant differences among TS‐treated groups (*P* < 0.05).

Abbrevations: BL, body length; BW, body weight; CON, control group; CW, carcass weight; MM, myocardial mass; TL, tibia length; TS, tail suspension; TS2, 2 week tail suspension group; TS4, 4 week tail suspension group.

### 3.2 Mouse food intake and water consumption

Food intake showed no significant differences among the three groups in the first (*P* = 0.297), second (*P* = 0.179) and fourth weeks (*P* = 0.357). However, water consumption showed significant differences in the first (*P* < 0.001) and second weeks (*P* = 0.012), suggesting that it might be necessary to control food intake and water consumption in animals in future research (Table 3).

**TABLE 3:** Effects of tail suspension on food intake and water consumption each week in mice.

Parameter	CON	TS2	TS4
FI in week 1 (g)	40.23 ± 4.62	41.23 ± 6.59	38.33 ± 4.35
FI in week 2 (g)	41.36 ± 7.32	41.79 ± 4.90	38.32 ± 4.28
FI in week 3 (g)	40.99 ± 7.51^a^	37.38 ± 4.90^a^	36.64 ± 2.22^b^
FI in week 4 (g)	41.49 ± 6.88	43.51 ± 39.12	41.26 ± 2.58
WC in week 1 (g)	56.41 ± 6.21^a^	55.22 ± 12.14 ^a^	37.71 ± 10.91^b^
WC in week 2 (g)	58.51 ± 7.31^a^	60.03 ± 12.63^a^	48.74 ± 12.58^b^
WC in week 3 (g)	64.43 ± 18.95^a^	70.9 ± 22.41^a^	56.36 ± 11.31^b^
WC in week 4 (g)	64.24 ± 26.46^a^	73.73 ± 29.17^a^	56.14 ± 15.02^b^

*Note*: Values are means ± SD (*n* = 16). Different superscript letters indicate significant differences among tail suspension‐treated groups (*P* < 0.05).

Abbreviations: CON, control group; FI, food intake; TS2, 2 week tail suspension group; TS4, 4 week tail suspension group; WC, water consumption.

### 3.3 Ultrastructural changes in myocardial mitochondria

**FIGURE 1:**
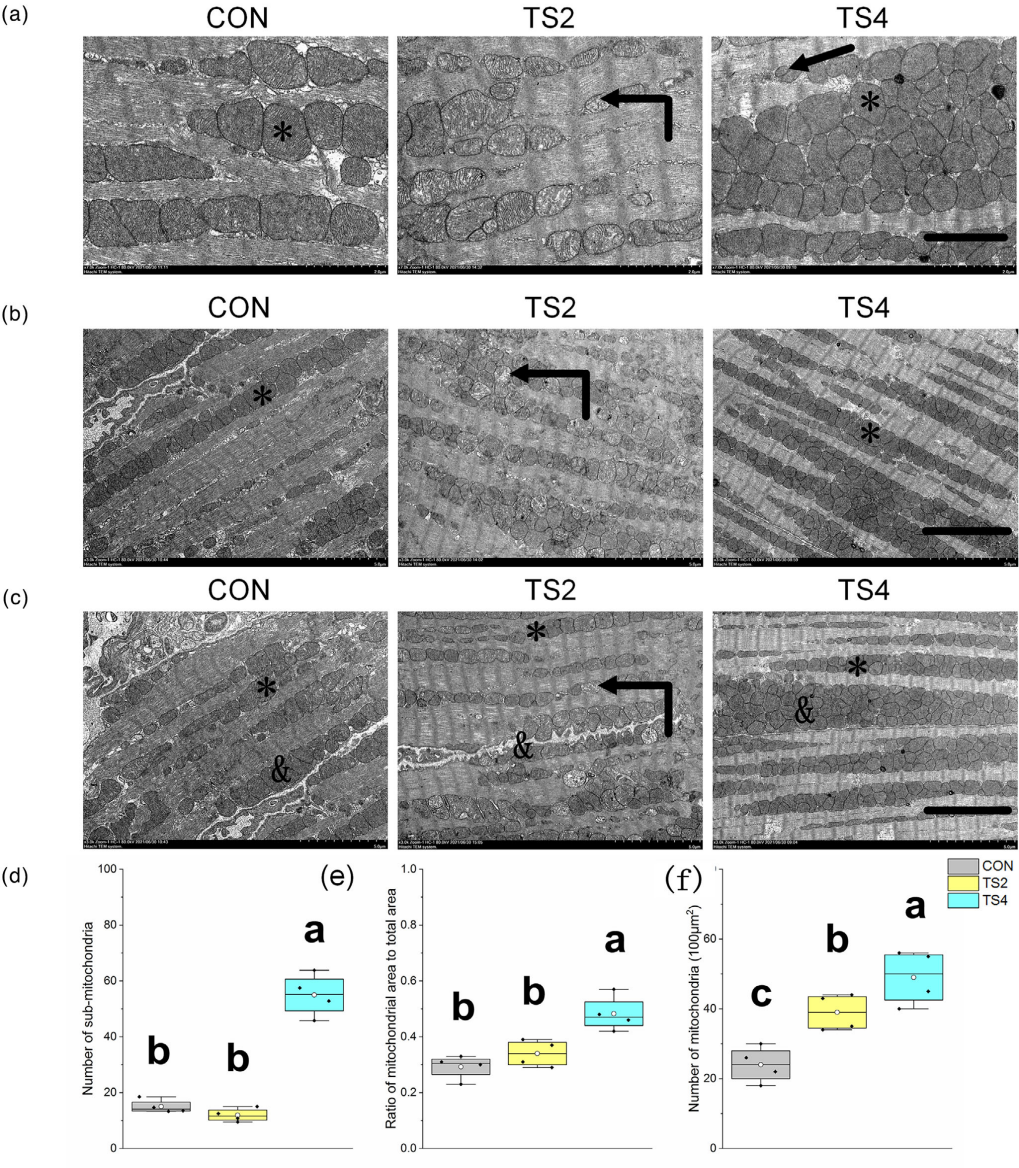
Changes in the number and area ratio of myocardial mitochondria in mice. (a) Ultrastructure of myocardial mitochondria in mice. Scale bar: 2 μm. Arrow shows smaller mitochondria in the TS4 group. (b) Intercellular ultrastructure of myocardial mitochondria in mice from three treatment groups. Scale bar: 5 μm. (c) Ultrastructure of myocardial sub‐mitochondria in mice from three treatment groups. Scale bar: 5 μm. *Normal intercellular mitochondria with clear cristae and intact membranes. In the TS2 group, mitochondria (curved arrow) were swollen and cristae disordered. ^&^Normal sub‐mitochondria with clear cristae and intact membranes. (d) Boxplot of number of sub‐mitochondria. (e) Ratio of mitochondrial area to total area. (f) Boxplot of number of mitochondria. Boxes represent upper and lower quartiles; middle horizontal line represents median; open circle represents average; lines extending from upper and lower ends represent upper and lower edges, respectively; asterisks represent extreme outliers; and points represent individual sample values. Six figures were analysed in each sample; four samples were analysed in each group. Different letters indicate significant differences among TS‐treated groups (*P* < 0.05). Abbreviations: CON, control group; TS2, 2 week tail suspension group; TS4, 4 week tail suspension group.

An interesting trend in mitochondrial morphology was observed among the three treatment groups. Specifically, swollen mitochondria and disrupted cristae were observed in the TS2 group. In contrast, the CON and TS4 groups showed smooth and intact mitochondrial membranes, with mitochondrial inner membrane‐filled cristae. The mitochondrial cristae were more compact in the TS4 group than in the CON group, and the mitochondria were smaller, exhibiting a complete structure and morphology (Figure 1a).

Most of the myocardial mitochondria were distributed between the muscle filaments (Figure 1b). The mitochondrial area to total area ratio was higher in the TS4 group than in the CON (*P* < 0.001) and TS2 (*P* = 0.048) groups (Figure 1e). The number of mitochondria followed the order TS4 > TS2 > CON (*P* < 0.001), and the number of normal mitochondria was similar in the CON and TS2 groups. In addition, mitochondria in the TS4 group were more densely distributed than in the other two groups (Figure 1f). Sub‐mitochondrial morphology was similar to that of intercellular mitochondria. The number of sub‐mitochondria was higher in the TS4 group than in the other groups (*P* < 0.04), suggesting that mitochondria were enriched under the sarcolemma (Figure 1d).

### 3.4 Changes in ultrastructure of myocardial nuclei and DNA fragmentation

**FIGURE 2:**
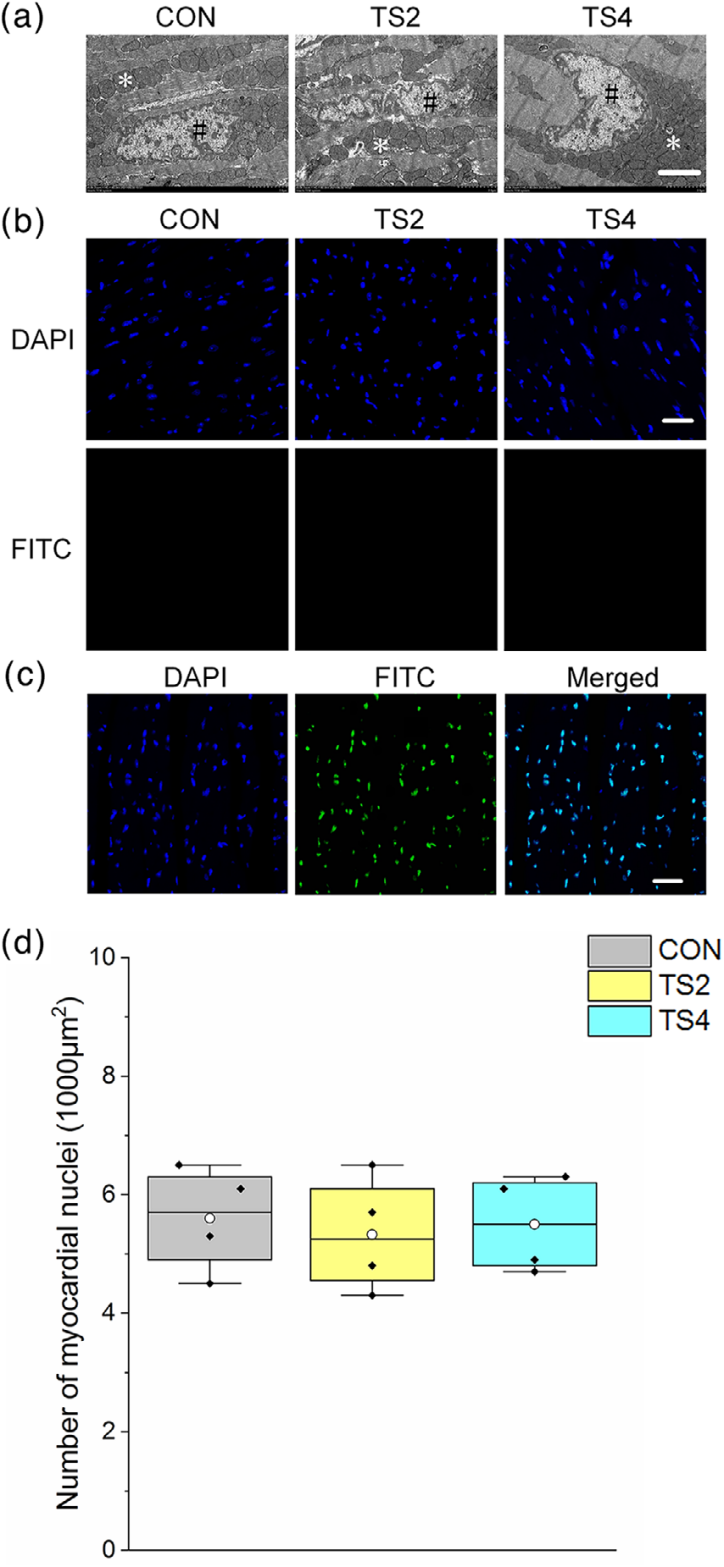
Ultrastructure of the myocardial nucleus and fluorescent terminal deoxynucleotidyl transferase biotin‐dUTP nick end‐labelling (TUNEL) staining of cardiac muscle in mice. (a) Ultrastructure of myocardial nucleus. Scale bar: 2 μm. Myocardial nuclei (#) are distributed between myofilaments. ^*^Normal intercellular mitochondria with clear cristae and intact membranes. (b) TUNEL staining of cardiac muscle in mice. Scale bar: 50 μm. Blue represents 4′6′‐diamidino‐2‐phenylindole (DAPI)‐stained nucleus; green represents TUNEL by FITC. (c) Positive control of TUNEL staining of cardiac muscle in mice. Scale bar: 50 μm. Blue represents DAPI‐stained nucleus; green represents TUNEL by FITC; and merged represents co‐localization of nucleus and TUNEL. (d) Boxplot of number of myocardial nuclei in mice. Boxes represent upper and lower quartiles; middle horizontal line represents median; open circle represents average; lines extending from upper and lower ends represent upper and lower edges, respectively; asterisks represent extreme outliers; and points represent individual sample values. Six figures were analysed in each sample; four samples were analysed in each group. Abbreviations: CON, control group; TS2, 2 week tail suspension group; TS4, 4 week tail suspension group.

Myocardial nuclei were mostly distributed between the myofilaments, with almost no chromatin agglutination or nuclear damage observed in the three groups (Figure 2a). Furthermore, no significant TUNEL reaction (DNA fragmentation) was observed in random myocardial sections of the three groups (Figure 2b,c). The number of nuclei also remained stable among the three groups (Figure 2d).

### 3.5 Changes in activity and protein levels in mitochondrial oxidative respiratory function‐related factors

**FIGURE 3:**
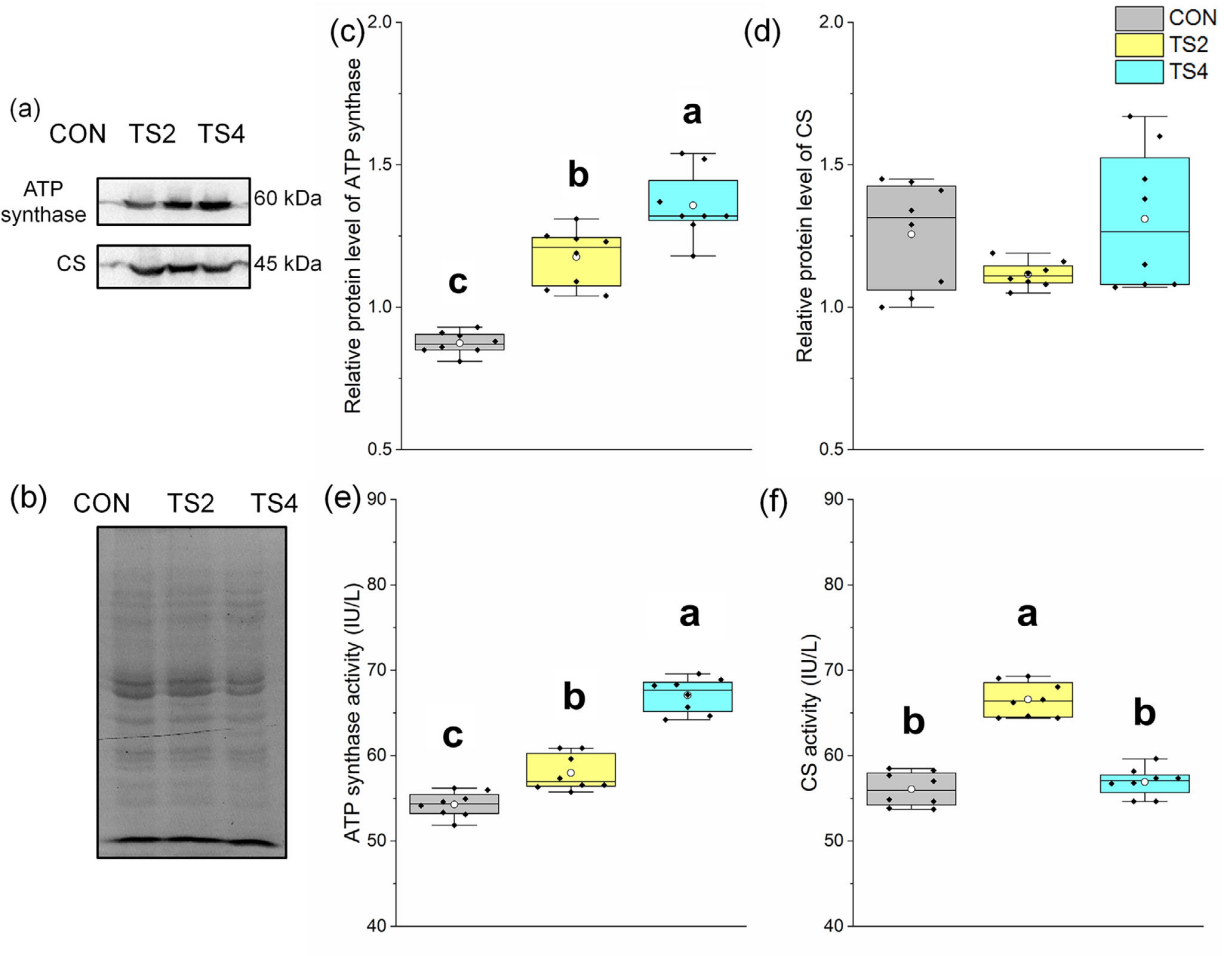
Protein and activity levels of myocardial mitochondrial oxidative respiratory function‐related factors in mice. (a) Representative immunoblots of myocardial ATP synthase and CS. (b) Representative polyacrylamide gel of total protein. (c) Relative protein level of ATP synthase. (d) Relative protein level of CS. (e) ATP synthase activity level. (f) Citrate synthase activity level. Boxes represent upper and lower quartiles; middle horizontal line represents median; open circle represents average; lines extending from upper and lower ends represent upper and lower edges, respectively; asterisks represent extreme outliers; and points represent individual sample values. *n* = 8. Different letters indicate significant differences among TS‐treated groups (*P* < 0.05). Abbreviations: CON, control group; CS, citrate synthase; TS2, 2 week tail suspension group; TS4, 4 week tail suspension group.

Representative western blot gels of ATP synthase and CS are shown in Figure 3a, and the total protein gel reference is shown in Figure 3b. The protein levels of ATP synthase followed the same order as that of ATP synthase activity (i.e., TS4 > TS2 > CON; *P* < 0.001; Figure 3c,d), indicating that the ability of mitochondria to produce ATP in the TS4 group was greater than that in the CON group.

Citrate synthase activity was significantly higher in the TS4 group than in the TS2 and CON groups (*P* < 0.001), but the protein level showed no significant differences among the three groups (Figure 3e,f).

### 3.6 Changes in levels of mitochondrial fission, autophagy and fusion‐related proteins

**FIGURE 4:**
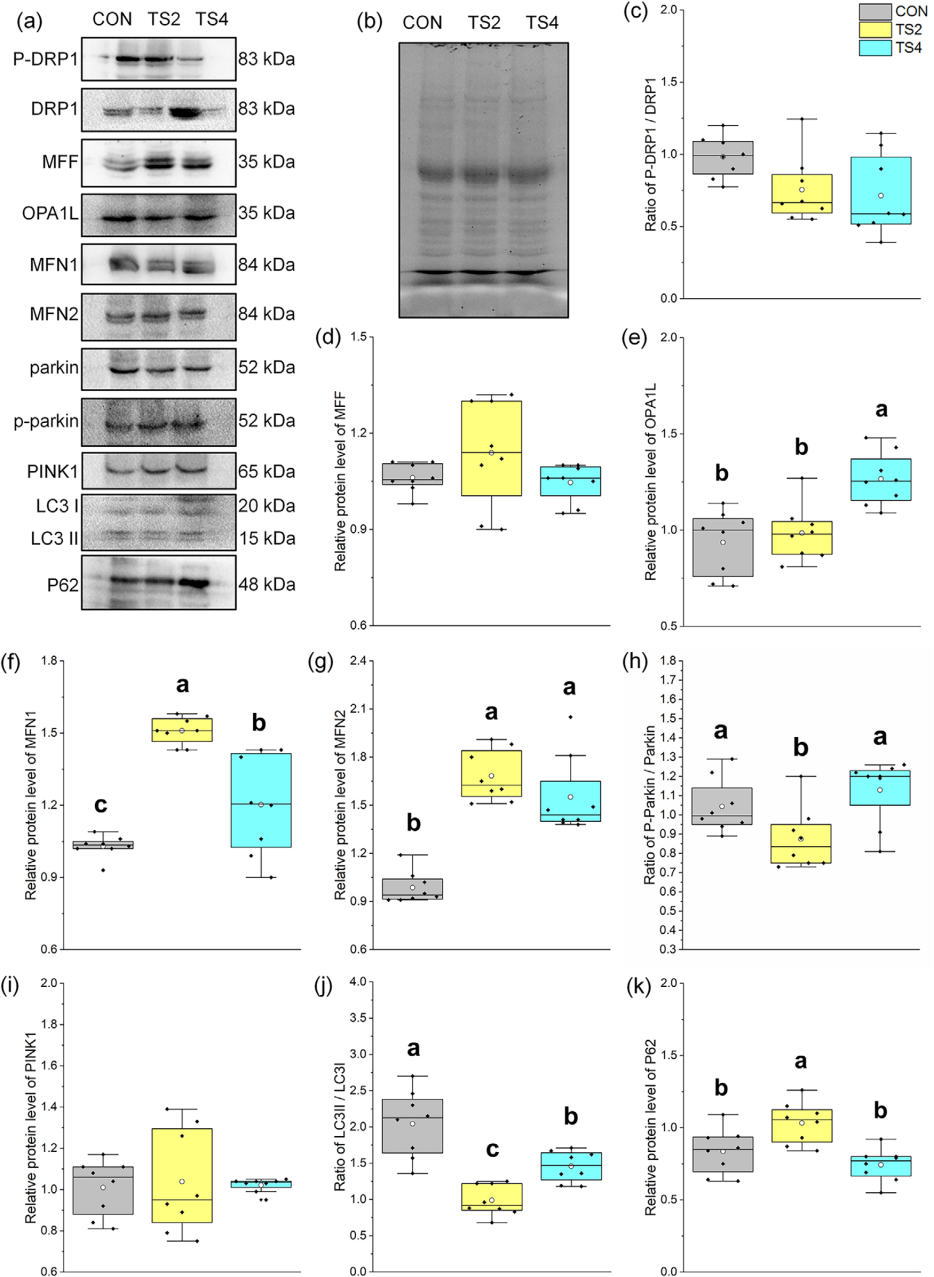
Protein levels of myocardial mitochondrial fission, fusion and autophagy‐related factors in mice. (a) Representative immunoblots of myocardial mitochondrial fission, fusion and autophagy‐related factors. (b) Representative polyacrylamide gel of total protein. (c) Ratio of P‐DRP1 to DRP1. (d) Relative protein level of MFF. (e) Relative protein level of OPA1L. (f) Relative protein level of MFN1. (g) Relative protein level of MFN2. (h) Ratio of p‐parkin to parkin protein level. (i) Relative protein level of PINK1. (j) Ratio of LC3II to LC3I. (k) Relative protein level of P62. Boxes represent upper and lower quartiles; middle horizontal line represents median; open circle represents average; lines extending from upper and lower ends represent upper and lower edges, respectively; asterisks represent extreme outliers; and points represent individual sample values. *n* = 8. Different letters indicate significant differences among TS‐treated groups (*P* < 0.05). Abbreviations: CON, control group; TS2, 2 week tail suspension group; TS4, 4 week tail suspension group.

Representative western blot gels of mitochondrial fission, autophagy and fusion‐related proteins are shown in Figure 4a, and the total protein gel reference is shown in Figure 4b. The phosphorylation ratio of DRP1 (Kashatus et al., 2015; Tong et al., 2020) and protein level of mitochondrial fission factor (MFF) did not show significant differences among the three groups, indicating that the level of mitochondrial fission remained stable (Figure 4c,d).

The phosphorylation ratio of parkin (*P* = 0.004) and ratio of LC3II to LC3I (*P* = 0.008) were higher in the TS4 group than in the TS2 group (Figure 4h–k), indicating an increase in mitochondrial autophagy in the TS4 group.

Representative western blot gels of optic atrophy 1 long subtype (OPA1L), MFN1 and MFN2 are shown in Figure 4a, and the total protein gel reference is shown in Figure 4b. The protein levels of these three factors were lowest (*P* < 0.001) in the CON group (Figure 4c–e), suggesting an increase in mitochondrial fusion in the TS groups compared with the CON group.

### 3.7 Changes in levels of apoptosis‐related proteins

**FIGURE 5:**
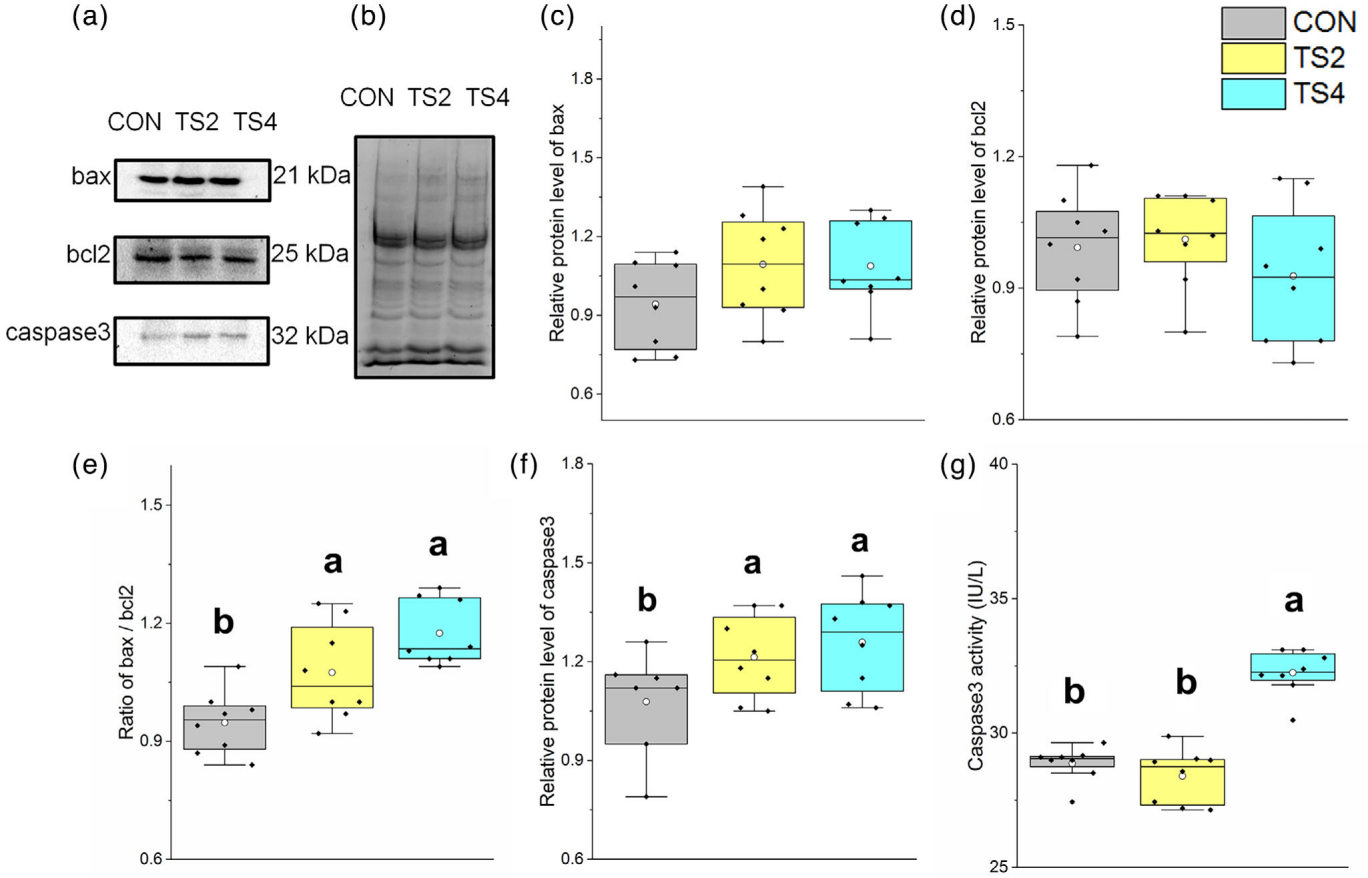
Protein levels of myocardial apoptosis‐related factors in mice. (a) Representative immunoblots of myocardial bax, bcl‐2 and caspase 3. (b) Representative polyacrylamide gel of total protein. (c) Relative protein level of bax. (d) Relative protein level of bcl‐2. (e) Ratio of bax to bcl‐2 protein level. (f) Relative protein level of caspase 3. (g) Caspase 3 activity level. Boxes represent upper and lower quartiles; middle horizontal line represents median; open circle represents average; lines extending from upper and lower ends represent upper and lower edges, respectively; asterisks represent extreme outliers; and points represent individual sample values. *n* = 8. Different letters indicate significant differences among TS‐treated groups (*P* < 0.05). Abbreviations: CON, control group; TS2, 2‐week tail suspension group; TS4, 4 week tail suspension group.

Representative western blot gels of bax, bcl‐2 and caspase 3 are shown in Figure 5a, and the total protein gel reference is shown in Figure 5b. The caspase 3 protein level and bax/bcl‐2 ratio increased (*P* < 0.001) in the two TS groups compared with that in the CON group (Figure 5e,g), suggesting an increase in the bax‐mediated myocardial apoptosis pathway.

## 4 DISCUSSION

After 4 weeks of TS treatment, body growth in mice was significantly inhibited, consistent with earlier research showing lower mouse weights after 2 and 4 weeks of TS compared with control animals of the same age (Loktev & Ogneva, 2019; Powers & Bernstein, 2004). The MM/TL ratio was also lower in the TS2 group than in the control group, suggesting that TS2 treatment might result in the loss of myocardial mass in mice. However, it was significantly higher in the TS4 group than in the TS2 group. Previous studies have confirmed that myocardial mass in mice begins to increase after 4 weeks of TS, probably owing to compensatory hypertrophy of cardiomyocytes caused by remodelling of the heart structure (Loktev & Ogneva, 2019; Suzuki et al., 2007), leading to the weakening of myocardial contractile force (Respress et al., 2014).

Of note, our results demonstrated that TS2 treatment caused degenerative changes in the mitochondria of the mouse myocardium, possibly leading to diminished mitochondrial function, whereas TS4 treatment appeared to relieve this change. This differs from previous reports on the adductor longus and soleus muscles of mice during space flight, showing loss of sub‐mitochondria, mitochondrial swelling and disruption of the cristae (Kim et al., 2007; Riley et al., 1992). The changes observed in our study suggest that the morphological recovery and increased number of myocardial mitochondria in the TS4 group might lead to enhanced mitochondrial function. Overall, myocardial mitochondrial morphology showed obvious degenerative changes in the TS2 group, whereas the myocardial mitochondria in the TS4 group not only recovered (with even denser mitochondrial cristae) but also increased in number.

Mitochondria play a crucial role in oxygen oxidation and energy supply, with ATP synthase and CS as key factors (Kramarova et al., 2008; Remington, 1992; Wiegand & Remington, 1986). Our results indicated that during TS treatment, the ATP‐producing capacity of mitochondria and aerobic oxidation might be stronger than that in the CON group. This might be related to the increase in the total number of myocardial mitochondria in the two TS groups of mice, which has important implications for changes in physiological state caused by blood redistribution during simulated weightlessness. Studies have shown that mitochondrial aerobic oxidation is accompanied by an increase in reactive oxygen species (Bae et al., 2011; Figueir et al., 2013; Kenny et al., 2017), which might lead to changes in mitochondrial morphology (Papa & Skulachev, 1997; Skulachev, 1996). Thus, the fragmentation and cavitation of mitochondrial cristae found in the TS2 group might be related to an increase in aerobic oxidation.

Ultrastructural observations of the myocardial nuclei showed no chromatin agglutination or nuclear membrane damage. DNA fragmentation, as the most direct evidence of apoptosis (Fu et al., 2016; Molpeceres et al., 2007), was observed, verifying the above findings. These results are similar to those of previous studies showing that apoptosis is not associated with myocardial atrophy (Chang et al., 2011).

We also analysed mitochondrial fission, fusion and autophagy‐related factors, which are crucial for the renewal of mitochondrial morphology (Fekkes et al., 2000; Michalska et al., 2016). Mitochondrial fusion and autophagy are the primary mechanisms by which damaged mitochondria are cleared (García‐Macia et al., 2019; Sarparanta et al., 2017; Wang & Wang, 2019). Although mitochondrial fission remained stable in the TS4 group compared with the TS2 group, several autophagic markers, including LC3 conversion and parkin phosphorylation, and mitochondrial fusion levels were higher in the TS4 group. Given that autophagy can aid in cellular clearage and restoration of mitochondrial homeostasis (García‐Macia et al., 2014; Santos‐Ledo et al., 2021), both mitophagy and fusion increase mitochondrial turnover. These factors contributed to the maintenance of mitochondrial morphology and increased number of mitochondria in the TS4 group.

**FIGURE 6:**
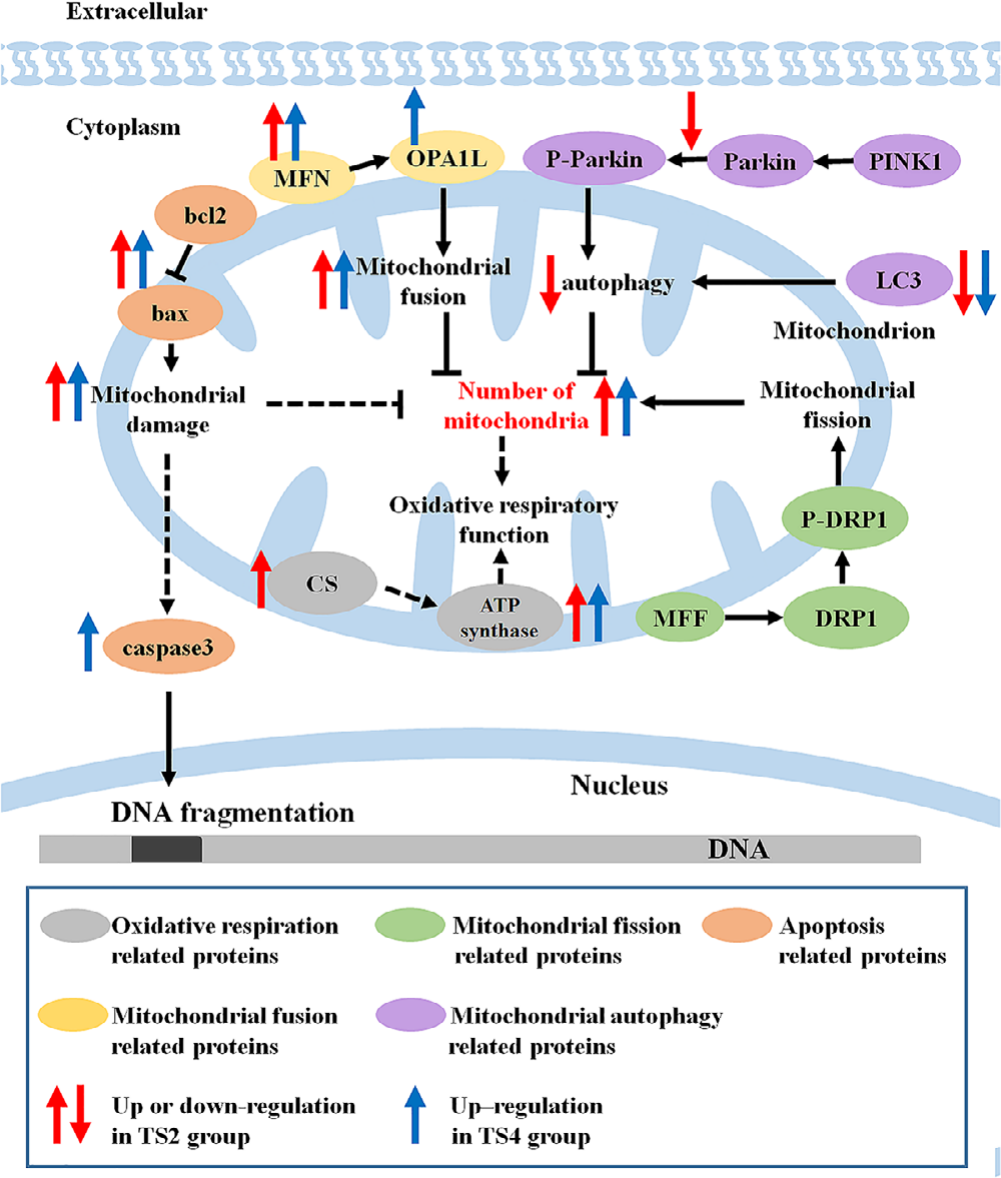
Graphical summary of study. Abbreviations: DRP1, dynamin‐related protein 1; MFF, mitochondrial fission factor; MFN, mitofusins; OPA1L, optic atrophy 1 long subtype; parkin, Parkinson's disease protein 2; PINK1, PTEN‐induced putative kinase 1; TS2, 2‐week tail suspension group; TS4, 4 week tail suspension group.

In summary, we explored the regulatory mechanisms related to the balance of apoptosis and mitochondrial fission, fusion and autophagy, in addition to the structure, number and function of myocardial mitochondria in mice during TS (Figure 6). Our findings indicate that TS2 alters the morphology of myocardial mitochondria in mice, but this change can be alleviated by TS4 treatment. The increase in mitochondrial autophagy and fusion promotes mitochondrial regeneration, which might explain the recovery of the morphology and number of mitochondria in the TS4 group. Moreover, the TS4 group exhibited a greater capacity for energy supply than the TS2 group, which might reflect an adaptive change in mice to simulated weightlessness.

## AUTHOR CONTRIBUTIONS

Zhe Wang, Xing‐Chen Wang and Ya‐Fei Chen conceived and designed the research; Zhe Wang, Xing‐Chen Wang, Ya‐Fei Chen, Ming‐Yue Jiang, Le Chen, Xiao‐Xuan Zhang, Xi‐Wei Liu and Ya‐Fei Chen performed the experiments; Zhe Wang analysed the data; Ya‐Fei Chen and Yong‐Zhen Feng interpreted the experimental results; Ya‐Fei Chen and Zhe Wang prepared the figures; Zhe Wang, Ya‐Fei Chen, Chuan‐Li Wang and Jin‐Hui Xu drafted the manuscript; Xing‐Chen Wang and Ya‐Fei Chen provided experimental guidance and suggestions for revision; Zhe Wang and Ya‐Fei Chen edited the manuscript and approved the final version of the manuscript.

## CONFLICT OF INTEREST

The authors declare no conflict of interest.

## Supporting information

Statistical Summary Document

## Data Availability

The datasets used or analysed during the study are included in the Supporting Information.
