# Supplementary material for: Loss and recovery of myocardial mitochondria in mice under different tail suspension time: Apoptosis and mitochondrial fission, fusion and autophagy
Source: Exp Physiol. 2023 Aug 11;108(9):1189–202. doi: 10.1113/EP090518 (PMC10988507; doi:10.1113/EP090518)
Supplement: Supplementary file 1 — Statistical Summary Document [file EPH-108-1189-s001.pdf]

The Journal of Physiology Statistical Summary Document

Manuscript Title: Loss and recovery of myocardial mitochondria in mice under different tail suspension time: apoptosis and mitochondrial fission, fusion, and autophagy

Authors: Zhe Wang, Xing-Chen Wang, Ya-Fei Chen, Chuan-Li Wang, Le Chen, Ming-Yue Jiang, Xi-Wei Liu, Xiao-Xuan Zhang, Yong-Zhen Feng, Jin-Hui Xu

Animal model used, if applicable: Kunming (KM) mice (*Mus musculus*).

Underlying hypothesis: This investigation tests the hypothesis that the morphology, number, and oxidative respiratory function of myocardial mitochondria would change in mice after different periods of TS. This investigation tests also hypothesized that these changes would be related to changes in apoptosis and mitochondrial fission, fusion, and autophagy.

Definitions of 'n':

Question 1: n = Water intake, food intake and myocardial mass coefficient, from at least 16 mice each group (CON, TS2 and TS4 groups), showed in table 1 and 2.
Question 2: n = individual mitochondria and TUNEL staining in mice myocardial slices, from at least 4 mice each group (CON, TS2 and TS4 groups).
Question 3: n = Protein and activity levels of myocardial mitochondrial homeostasis related factors, from at least 8 mice each group (CON, TS2 and TS4 groups).

Statistical summary table:

Experimental question number*	Finding/ conclusion	Experimental location/ variable<br>e.g. muscle, neocortex or genotype	Mean value<br>(or other summary statistic)	SD	n val.	P**	Units	Data comparisons<br>e.g. WT vs KO	Statistical test	Any other variable<br>e.g. subjects' age or sex	Figure/ table in which data are presented	Comments<br>e.g. observation
Changes in number and area ratio of myocardial mitochondria	Number of myocardial sub-mitochondria	Tail suspension Duration	15	2.4	4	0.001	fold	CON vs TS2 vs TS4	One-way analysis of variance	no	1	observation
			11.9	2.3	4							
			54.9	3.7	4							
	Ratio of mitochondria		0.29	0.04	4	0.002	fold	CON vs TS2 vs TS4		no	1	observation
			0.34	0.05	4							

	l area to total area	Tail suspension Duration	0.48	0.06	4				One-way analysis of variance			
	mitochondrial number	Tail suspension Duration	24	5.1	4	0.001	fold	CON vs TS2 vs TS4	One-way analysis of variance	no	1	observation
			39	5.2	4							
			49	7.7	4							
Number of myocardial nuclei	Number of myocardial nuclei	Tail suspension Duration	5.6	0.88	4	0.909	NA	CON vs TS2 vs TS4	One-way analysis of variance	no	1	observation
			5.3	0.97	4							
			5.5	0.81	4							
Protein and activity levels of myocardial mitochondrial oxidative respiratory function-related factors	Relative protein level of ATP synthase	Tail suspension Duration	0.87	0.04	8	0.001	fold	CON vs TS2 vs TS4	One-way analysis of variance	no	1	observation
			1.17	0.09	8							
			1.35	0.11	8							
	Relative protein level of CS	Tail suspension Duration	1.25	0.18	8	0.043	fold	CON vs TS2 vs TS4	One-way analysis of variance	no	1	observation
			1.11	0.04	8							
			1.31	0.24	8							
	ATP synthase activity level	Tail suspension Duration	54.2	1.47	8	0.001	fold	CON vs TS2 vs TS4	One-way analysis of variance	no	1	observation
			57.9	2.13	8							
			67.1	2.01	8							
	CS activity level		56	2	8	0.001	fold	CON vs TS2 vs TS4		no	1	observation
			66.6	2.1	8							

		Tail suspension Duration	56.9	1.7	8				One-way analysis of variance			
Protein levels of myocardial mitochondrial fission, fusion and autophagy - related factors	Ratio of P-DRP1 to DRP1	Tail suspension Duration	0.98	0.14	8	0.027	fold	CON vs TS2 vs TS4	One-way analysis of variance	no	1	observation
			0.75	0.23	8							
			0.71	0.28	8							
	Relative protein level of MFF	Tail suspension Duration	1.06	0.04	8	0.158	NA	CON vs TS2 vs TS4	One-way analysis of variance	no	1	observation
			1.13	0.16	8							
			1.04	0.06	8							
	Relative protein level of OPA1L	Tail suspension Duration	0.93	0.16	8	0.001	fold	CON vs TS2 vs TS4	One-way analysis of variance	no	1	observation
			0.98	0.14	8							
			1.26	0.13	8							
	Relative protein level of MFN1	Tail suspension Duration	1.03	0.05	8	0.001	fold	CON vs TS2 vs TS4	One-way analysis of variance	no	1	observation
			1.51	0.05	8							
			1.2	0.21	8							
	Relative protein level of MFN2	Tail suspension Duration	0.98	0.09	8	0.001	fold	CON vs TS2 vs TS4	One-way analysis of variance	no	1	observation
			1.68	0.15	8							
			1.55	0.24	8							
	The ratio of p-parkin to		1.04	0.14	8	0.043	fold	CON vs TS2 vs TS4		no	1	observation
			0.87	0.15	8							

	parkin protein level	Tail suspension Duration	1.12	0.16	8				One-way analysis of variance			
	Relative protein level of PINK1	Tail suspension Duration	1.01	0.13	8	0.732	NA	CON vs TS2 vs TS4	One-way analysis of variance	no	1	observation
			1.03	0.25	8							
			1.02	0.03	8							
	Ratio of LC3II to LC3I	Tail suspension Duration	2.04	0.46	8	0.001	fold	CON vs TS2 vs TS4	One-way analysis of variance	no	1	observation
			0.98	0.21	8							
			1.45	0.21	8							
	Relative protein level of P62	Tail suspension Duration	0.83	0.15	8	0.001	fold	CON vs TS2 vs TS4	One-way analysis of variance	no	1	observation
			1.03	0.14	8							
			0.74	0.11	8							
Protein levels of myocardial apoptosis-related factors	Relative protein level of bax	Tail suspension Duration	0.94	0.16	8	0.112	NA	CON vs TS2 vs TS4	One-way analysis of variance	no	1	observation
			1.09	0.2	8							
			1.08	0.17	8							
	Relative protein level of bcl-2	Tail suspension Duration	0.99	0.13	8	0.342	NA	CON vs TS2 vs TS4	One-way analysis of variance	no	1	observation
			1.01	0.11	8							
			0.92	0.16	8							
	The ratio of bax to bcl-2		0.94	0.08	8	0.001	fold	CON vs TS2 vs TS4		no	1	observation
			1.07	0.12	8							

		Tail suspension Duration	1.17	0.08	8				One-way analysis of variance			
	Relative protein level of caspase3	Tail suspension Duration	1.05	0.15	8	0.001	fold	CON vs TS2 vs TS4	One-way analysis of variance	no	1	observation
			1.21	0.12	8							
			1.25	0.15	8							
	Caspase3 activity level	Tail suspension Duration	28.8	0.65	8	0.012	fold	CON vs TS2 vs TS4	One-way analysis of variance	no	1	observation
			28.4	1.01	8							
			32.2	0.85	8							

*You may use multiple lines for the same question to indicate multiple comparisons

** Authors may wish to make the text bold where p is considered significant against a stated confidence limit.
